# Germline genetic variation in prostate susceptibility does not predict outcomes in the chemoprevention trials PCPT and SELECT

**DOI:** 10.1038/s41391-019-0181-y

**Published:** 2019-11-27

**Authors:** Mahbubl Ahmed, Chee Goh, Edward Saunders, Clara Cieza-Borrella, the PRACTICAL consortium, Zsofia Kote-Jarai, Fredrick R. Schumacher, Ros Eeles

**Affiliations:** 1 The Institute of Cancer Research, Royal Marsden Hospital, NHS Foundation Trust, 123 Old Brompton Road, London, SW7 3RP UK; 2 Department of Epidemiology and Biostatistics, Case Western Reserve University; Seidman Cancer Center, University Hospitals, Cleveland, OH USA

**Keywords:** Cancer genetics, Prostate cancer

## Abstract

**Background:**

The development of prostate cancer can be influenced by genetic and environmental factors. Numerous germline SNPs influence prostate cancer susceptibility. The functional pathways in which these SNPs increase prostate cancer susceptibility are unknown. Finasteride is currently not being used routinely as a chemoprevention agent but the long term outcomes of the PCPT trial are awaited. The outcomes of the SELECT trial have not recommended the use of chemoprevention in preventing prostate cancer. This study investigated whether germline risk SNPs could be used to predict outcomes in the PCPT and SELECT trial.

**Methods:**

Genotyping was performed in European men entered into the PCPT trial (*n* = 2434) and SELECT (*n* = 4885). Next generation genotyping was performed using Affymetrix® Eureka™ Genotyping protocols. Logistic regression models were used to test the association of risk scores and the outcomes in the PCPT and SELECT trials.

**Results:**

Of the 100 SNPs, 98 designed successfully and genotyping was validated for samples genotyped on other platforms. A number of SNPs predicted for aggressive disease in both trials. Men with a higher polygenic score are more likely to develop prostate cancer in both trials, but the score did not predict for other outcomes in the trial.

**Conclusion:**

Men with a higher polygenic risk score are more likely to develop prostate cancer. There were no interactions of these germline risk SNPs and the chemoprevention agents in the SELECT and PCPT trials.

## Introduction

Prostate cancer accounts for a quarter of cancer diagnoses in men in the UK and is the fourth most common cancer worldwide with an estimated 1.1 million men diagnosed in 2012 [1]. Screening strategies have not led to their routine clinical use in daily practice, due to an over-diagnosis of indolent cancers [2].

Due to its biology, prostate cancer is ideally suited target for chemoprevention because of its step-wise biological development. Prostate cancer may have precursor lesions such as atypical small acinar proliferation and high grade prostatic intraepithelial neoplasia which may appear many years before cancer is diagnosed. This high risk population with these precursor lesions could be targeted for chemoprevention [3]. Prostate cancer also has a long latent period from its development to its eventual clinical manifestation. Chemoprevention could also be used to prevent these early malignant lesions developing further and therefore many men could be spared diagnostic procedures and potentially toxic systemic treatments [4].

It is thought that the risk of prostate cancer development could be potentially modifiable by dietary and other lifestyle factors. Large epidemiological studies have shown that migrants who have moved from areas of low prostate cancer incidence such as Korea and Japan to the USA have developed prostate cancer rates similar to their native inhabitants [5, 6]. Environmental factors are attributable to this change of risk and some of these could be potentially modifiable by dietary changes [6]. There are also causal links between androgen exposure and the development of prostate cancer, which is also potentially modifiable [7]. The need to answer the question of the role of chemoprevention in prostate cancer was the aim of two large randomised controlled trials: The Prostate cancer Prevention Trial (PCPT) and The Selenium and Vitamin E Cancer Prevention Trial (SELECT). The biological rationale for the agents used in the trial, trial schema and results can be found in more detail elsewhere [8, 9]. In summary, 18,880 men were eligible and randomised on the PCPT to receive placebo or finasteride. The results of the PCPT trial showed that prostate cancer was detected in 803 of the 4368 men in the finasteride group and 1147 of the 4692 men in the placebo group who had data for analyses. There was a 24.8% reduction in prevalence over the 7-year period. Tumours with a Gleason score 7–10 were more common in the finasteride group (280 of 757 tumours (37.0%)), than in the placebo group (237 of 1068 tumours (22.2%)) (*P* < 0.001 for the comparison between groups) [8]. The long-term results of the PCPT trial showed that the 10 year survival from prostate cancer was equivalent between the 2 groups [10].

In the SELECT trial, 35,533 men were randomised to receive selenium or vitamin E, or both or neither. The SELECT trial was closed early because the interim analysis showed that neither selenium, vitamin E, nor the combination prevented prostate cancer. The long-term results of the SELECT trial showed that men taking vitamin E had increased incidence of prostate cancer compared with placebo, which was statistically significant [9]. Genetic variations influencing the effects of these chemoprevention agents have been reported from the PCPT and SELECT trials [11–14]. However these studies have only used a candidate gene approach to look for association in comparison with our study which has followed up validated risk single nucleotide polymorphisms (SNPs).

At the time of undertaking these analyses, 100 germline risk SNPs had been identified in genome wide association studies [15, 16]. The 100 SNPs account for ~33% of familial risk of prostate cancer [16]. If polygenic risk scores are calculated, the top 10% of men in the highest risk stratum have an ~2.9-fold relative risk of prostate cancer compared with the average of the population, whilst the top 1% of men have a 5.7-fold relative risk in comparison with the population average [16]. If these polygenic SNP scores could be incorporated into screening with the addition of known risk factors (age, race, and family history (FHx)), then men with the highest risk of prostate cancer development could be targeted for more intensive screening or intervention.

Traditional genotyping technologies are currently not suited to provide fast accurate outputs that could be used in prospective clinical trials [17]. Genotype calling from next generation sequence techniques may be able to overcome these issues and therefore be more suited for everyday clinical use [18]. We have worked with Eureka Genomics (Hercules, CA; previously affymetrix now ThermoFisher Scientific) to design and validate a next generation genotyping assay in these analyses.

While the functional role of how these SNPs influence cancer predisposition is under investigation, evidence suggests that this may be through various pathways [19]. Therefore, the hypothesis underlying this study was that common genetic variants involved in prostate cancer predisposition may have a role in tumour formation but also influence the effects of the chemoprevention agents. In breast cancer, evidence has shown that chemoprevention with tamoxifen reduces the risk of contralateral breast cancer for those women who have variants in the *BRCA2* gene [20]. The aim of this study was to investigate the association of prostate cancer germline risk SNPs and their influence on the chemoprevention agents in the PCPT and SELECT trials.

## Materials and methods

### Patients

The details of the PCPT study can be found in more detail elsewhere [8]. Informed consent as obtained from all patients. In summary, healthy men aged 55 years or older, with a normal digital rectal examination (DRE), American Association Urology Score of less than 20 and no clinically significant co-morbidity were entered into the trial. The prostate specific antigen (PSA) on entry into the trial was required to be 3.0 ng/ml or lower. Men were randomised to receive Finasteride or placebo. The men underwent annual DRE, and measurements of PSA. If the DRE was suspicious for cancer or the adjusted PSA > 4.0 ng/ml, then these men were recommended to undergo a prostate biopsy. If these investigations were normal or biopsy was negative, men were followed up for a total of 7 years and an end of study biopsy was performed. The end of study biopsy identified additional prostate cancers among men with neither a sspicious DRE or elevated PSA and confirmed that the controls did not have prostate cancer. A total of 18,880 eligible men were randomised into the trial. Our institution received DNA samples from 2434 cases and controls from the PCPT study.

The details of the SELECT trial can found in more detail elsewhere [21]. Healthy men entered the study and were aged 55 years or over (Afro-American men were aged 50 or older), had a normal DRE and a PSA ≤ 4 ng/ml. Participants were randomised into one of four groups: Selenium alone, vitamin E alone, selenium and vitamin E or placebo. Men were followed every 6 months and suggested to have an annual DRE and PSA. Men were recommended to undergo PSA and DRE testing and prostate biopsy based on local care guidelines. Upon a diagnosis of prostate cancer, men were monitored annually.

A total of 35,553 men were randomised into the trial. Our institution received DNA samples from 4885 men, which were samples that were selected to be analysed as part of a case-cohort study.

### Laboratory methods, genotyping and quality control

DNA was collected by white blood cells from participants in the PCPT and SELECT trials and extracted at the National Cancer Institute [22]. Thirty microlitres of DNA was received at The Institute of Cancer Research; 5 µl was sent for genotyping for the purpose of this analysis.

At the time of designing the genotyping assay the latest 100 SNP panel was used [23]. Supplementary Table 2 lists the 100 known prostate cancer susceptibility SNPs at that time.

The 100 SNP panel was developed by Affymetrix®, now a part of ThermoFisher Scientific. The samples were genotyped using the Affymetrix® Eureka™ Genotyping protocols. The Eureka genotyping assay is a ligation-dependent PCR reaction, which uses interrogation site bar codes contained within the ligation probes, as well as sample index bar codes added during the amplification step. Next generation sequencing libraries were created and short cycle sequence data were generated from the prepared libraries. Analysis software was used to tabulate the number of reads that contain each combination of sample, locus and allele bar code (as appropriate). The genotype was determined by in-house software using Affymetrix® Eureka™ protocols.

In order to validate this new genotyping technique two methods were used. Firstly, known genotypes of overlapping samples from the iCOGs custom array and the Affymetrix panel were contrasted [24]. Secondly the observed minor allele frequencies from the Affymetrix panel were compared with the genotypes of overlapping samples on a custom high-throughput array by case and control status [25]. The minor allele frequencies between the two techniques were highly correlated (*r*^2^ > 0.99).

Standard quality control measures were applied to remove variants with missing rates > 10% or displayed genotype frequency deviating from those expected under Hardy–Weinberg equilibrium (*P* < 0.05). Samples with less than 90% genotyping rate were also removed (Table 1).

**Table 1:** Quality control for both studies

	PCPT	SELECT
Number of SNPs genotyped	98	98
Total number of DNA samples received	2435	4789
European	2089	3512
>10% Genotype missing for individual lost		
Cases	0	47
Controls	0	67
SNP lost with less than 90% genotyping rate	5	9
Hardy–Weinberg equilibrium SNPs lost (*P* < 0.001)	0	0

### Measured outcome

On entry into the PCPT and SELECT trials, phenotypic information was collected on all participants; further detailed information on the phenotypic information collected can be found in the individual trial protocols [26, 27].

Men who self-reported to be of European origin were only included in this study as the vast majority of the germline SNPs used in this analysis were discovered in GWAS from populations of European descent. FHx was defined as men who had one or more first degree relatives affected with prostate cancer. Biopsies that were positive for prostate cancer were reviewed by the local pathologist at the participating centre, and for the PCPT trial and also reviewed centrally [8, 21]. High-grade prostate cancer (non-indolent prostate cancer) was defined as a Gleason score ≥ 7. A summary of basic phenotype information for the DNA received from PCPT and SELECT can be found here (Table 2).

**Table 2:** Patient characteristics (FHx—family history)

	PCPT		SELECT			
	Finasteride	Placebo	Placebo	Selenium	Vitamin E	Selenium & vitamin E
Numbers						
Total	833	1256	845	816	908	829
Cases	428	616	352	330	393	346
Control	405	640	493	486	515	483
Age						
Mean age of entry trial (SD)	64.4 (5.7)	63.6 (5.6)	63.9 (6.0)	63.8 (6.0)	64.0 (6.1)	63.7 (6.2)
Mean age of Ca Dx (SD)	69.9 (5.6)	69.7 (5.7)	67.1(5.8)	63.8 (6.0)	67.6 (6.1)	67.2 (6.2)
BMI (kg/m^2^)	27.5	27.4	NA	NA	NA	NA
FHx positive	195	263	180	149	188	178
Gleason ≤ 6	266	470	197	182	221	202
Gleason ≥ 7	157	138	123	115	143	114

### Statistical analysis

A polygenic risk score was calculated by summing the genotype dosage for all variants for an individual. The log-odds ratios used to weight the risk score were taken from the OncoArray Meta-analysis [28]. Two types of risk score were calculated:

Non-weighted, for patient *i*:${\mathrm{risk}\,\mathrm{score}_i} = \mathop {\sum}\nolimits_1^j {G_{ij}}$

Weighted, for patient *i*: ${\mathrm{weighted}\,\mathrm{risk}\,\mathrm{score}_i} = \mathop {\sum}\nolimits_1^j {\beta _j} G_{ij}$

*j* = variants 1–100

*β*_*j*_ = is the per allele log-odds ratio for the risk of prostate cancer associated with variant *j*

*G* = risk allele dosage

Within each cohort of both trials age and body mass index (BMI) were equally distributed. Logistic regression was used to test the association of case/control and Gleason score and the polygenic risk score. FHx was also used as a covariate in the analyses. The polygenic risk score was divided into quartiles for the logistic regression model and interaction tested. For each individual SNP the *χ*^2^ test was used to test the association with overall prostate cancer and sub-strata defined by Gleason grade. Statistical significance was determined at a level of *P* < 0.05. All analyses were performed in the statistical package R 3.2.2 [29].

### Power calculations

The power was calculated using the MAF and OR from the Oncoarray paper which was the largest GWAS performed to date [28]. A significance level of *α* = 0.05 was used and calculated using a genetic power calculator [30]. The results can be seen in Supplementary Table 1. The power ranged for PCPT (5–96%) and SELECT (5–99%). The power was greater in the SELECT trial as there were greater number of participants the PCPT. Overall the study was underpowered however there was a correlation between those with a high power and the signal seen of the SNP in PCPT/SELECT. A multiple comparison adjustment was not performed as known variants were analysed in PCPT/SELECT. Power was not calculated by randomisation arm as the power would have been too low.

## Results

The characteristics of the participants of the trial are summarised in Table 2. Of the 100 SNPs, 98 SNPs were designed successfully. Non-Europeans were removed from the analysis and summary of the quality control is shown in Table 3.

**Table 3:** Showing the significant single SNP association for the development of prostate cancer and nearby genes in PCPT trial

Trial arm	Chromosome	SNP	*P* value	Odds ratio	Nearby genes
Placebo	3	rs7611694	0.04367	0.8473	*SIDT1*
Placebo	7	rs12155172	0.007637	1.292	*SP8*
Placebo	8	rs6983267	0.00198	0.78	*N/A*
Placebo	17	rs1859962	0.003917	1.26	*N/A*
Finasteride	2	rs7584330	0.03174	0.7799	*MLPH*
Finasteride	8	rs16901979	0.008784	2.28	*N/A*
Finasteride	17	rs4430796	9.64E-05	1.489	*HNF1B*
Finasteride	17	rs684232	0.03556	1.241	*VPS53, FAM57A*
Finasteride	17	rs1859962	0.01778	1.262	*N/A*
Finasteride	18	rs7241993	0.01109	0.7619	*SALL3*
Finasteride	22	rs5759167	0.01402	0.7852	*BIL/TTLL1*

**Fig. 1:**
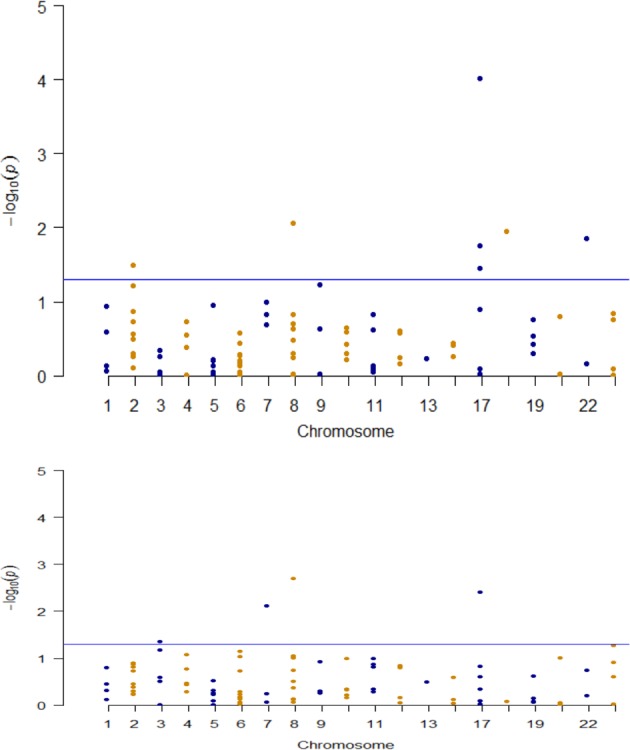
Manhattan plot showing the single SNP association of the placebo (above) and finasteride (below) arms in PCPT. Blue line represents significance level *P* = 0.05

**Fig. 2:**
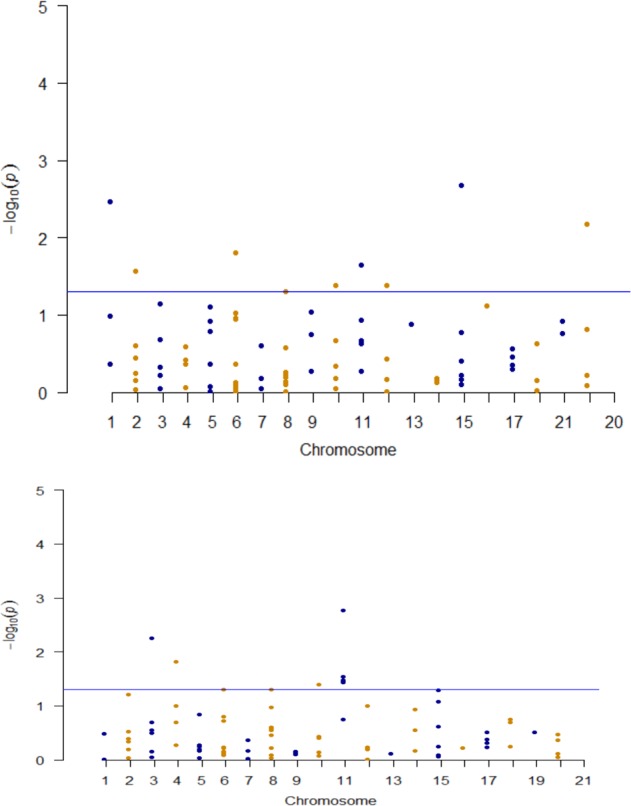
Manhattan plot showing the single SNP association of the placebo (above) and selenium (below) arms in SELECT. Blue line represents significance level *P* = 0.05

**Fig. 3:**
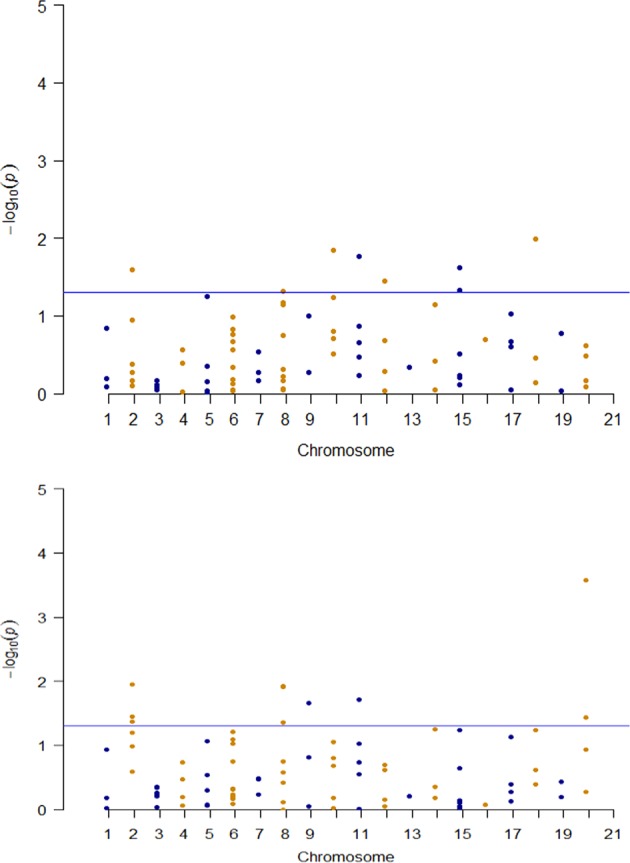
Manhattan plot showing the single SNP association of the vitamin E (above) and selenium and vitamin E (below) arms in SELECT. Blue line represents significance level *P* = 0.05

The single SNP association in both trials shows multiple SNPs that are associated with developing prostate cancer at a pre-defined significance level of *P* < 0.05 (Figs 1–3 and Tables 3 and 4). In the PCPT trial rs4430796 is associated in the finasteride arm with development of prostate cancer; the SNP resides near the gene *HNF1B*. There are also multiple SNPs which are significant for the association with high grade and low grade Gleason score (Table 5). In the PCPT trial as the polygenic risk score increases the beta predicting prostate cancer outcome increases and this is statistically significant. With the addition of Finasteride the beta reduces but it is not statistically significant (Table 6). In the SELECT trial the polygenic risk score did not predict cancer outcome except for those men who are in group 4 of the weighted risk score. There was no interaction between the interventions in the PCPT and SELECT trials and the polygenic risk score.

**Table 4:** Showing the significant single SNP association for the development of prostate cancer and nearby genes in SELECT trial

Trial arm	CHR	SNP	*P*	OR	Nearest gene
Placebo	1	rs636291	0.003377	0.6929	*PEX14*
Placebo	2	rs3771570	0.02715	1.404	*FARP2*
Placebo	6	rs4713266	0.01558	0.7583	*TNS3*
Placebo	8	rs1512268	0.05006	1.252	*NKX3.1*
Placebo	10	rs2252004	0.04196	0.683	*N/A*
Placebo	11	rs11214775	0.02261	0.7473	*HTR3B*
Placebo	12	rs1270884	0.04148	0.7919	*TBX5*
Placebo	17	rs1859962	0.002107	1.422	*N/A*
Placebo	23	rs5919432	0.006584	1.712	*AR*
Selenium	2	rs12621278	0.0256	0.563	*ITGA6*
Selenium	5	rs2242652	0.05695	0.7398	*TERT*
Selenium	8	rs2928679	0.04767	1.268	*SLC25A37*
Selenium	10	rs10993994	0.01418	1.345	*MSMB*
Selenium	10	rs2252004	0.05719	1.465	*N/A*
Selenium	11	rs1938781	0.0172	0.699	*FAM111A*
Selenium	12	rs80130819	0.03519	0.6222	*N/A*
Selenium	17	rs1859962	0.04747	1.267	*N/A*
Selenium	20	rs6062509	0.01023	0.7144	*ZGPAT*
Vit E	3	rs2660753	0.00565	1.659	*N/A*
Vit E	4	rs7679673	0.01517	0.7443	*TET2*
Vit E	6	rs2273669	0.04909	1.383	*ARMC2, SESN1*
Vit E	8	rs1512268	0.04919	1.261	*NKX3.1*
Vit E	10	rs10993994	0.03971	1.28	*MSMB*
Vit E	11	rs11214775	0.001709	0.661	*HTR3B*
Vit E	11	rs1938781	0.02891	0.7233	*FAM111A*
Vit E	11	rs7127900	0.03343	1.365	N/A
Vit E	11	rs7931342	0.03694	0.7825	N/A
Vit E	17	rs138213197	0.05212	3.532	N/A
Sel + Vit E	2	rs12621278	0.01127	0.5422	*ITGA6*
Sel + Vit E	2	rs10187424	0.03605	1.264	*GGCX/VAMP8*
Sel + Vit E	2	rs11902236	0.0422	1.275	*TAF1B:GRHL1*
Sel + Vit E	8	rs1447295	0.0118	1.556	N/A
Sel + Vit E	8	rs620861	0.01228	0.7508	N/A
Sel + Vit E	8	rs12543663	0.04453	1.273	N/A
Sel + Vit E	9	rs1571801	0.02191	0.7455	*DAB21P (aggressive SNP)*
Sel + Vit E	11	rs11568818	0.01959	0.7711	*MMP7*
Sel + Vit E	14	rs8014671	0.05637	1.237	N/A
Sel + Vit E	17	rs1859962	0.05754	1.234	N/A
Sel + Vit E	20	rs2427345	0.05815	0.8051	*GATAS, CABLES2*
Sel + Vit E	23	rs5945619	0.000263	1.808	*NUDT11*
Sel + Vit E	23	rs2405942	0.0367	0.6727	*SHROOM2*

**Table 5:** Showing the significant single SNP association of high and low grade Gleason score and nearby genes in PCPT and SELECT trial

Trial	CHR	SNP	*P*	OR	Nearest gene
SELECT	1	rs1218582	0.2969	0.8991	N/A
SELECT	1	rs636291	0.000788	0.6965	*PEX14*
PCPT	2	rs721048	0.02	1.487	*EHBP1*
SELECT	2	rs12621278	0.009552	0.563	*ITGA6*
SELECT	2	rs3771570	0.02863	1.35	*FARP2*
PCPT	3	rs2055109	0.01	0.6528	N/A
SELECT	3	rs2660753	0.00946	1.505	N/A
SELECT	3	rs10934853	0.01466	1.316	N/A
PCPT	5	rs2853676	0.04	1.368	N/A
PCPT	5	rs13190087	0.01	2.398	N/A
SELECT	5	rs2242652	0.02988	0.7401	*TERT*
PCPT	6	rs2273669	0.02	1.535	*ARMC2, SESN1*
SELECT	6	rs2273669	0.03273	1.324	*ARMC2, SESN1*
SELECT	6	rs2273669	0.03308	1.355	*ARMC2, SESN1*
SELECT	7	rs12155172	0.0316	1.281	N/A
PCPT	8	rs620861	0.02	0.6856	N/A
PCPT	8	rs11135910	0.05	0.6722	*EBF2*
SELECT	8	rs12543663	0.04911	1.24	N/A
SELECT	8	rs1512268	0.02339	1.254	*NKX3.1*
SELECT	8	rs1447295	0.02999	1.387	*8q24*
SELECT	8	rs16901979	0.003904	2.109	N/A
PCPT	10	rs3850699	0.04	1.343	*TRIM8*
SELECT	10	rs10993994	0.008375	1.315	*MSMB*
SELECT	10	rs2252004	0.003301	0.6115	N/A
PCPT	11	rs7127900	0.04	0.6679	N/A
PCPT	11	rs1938781	0.04	1.386	*FAM111A*
SELECT	11	rs1938781	0.01914	0.7441	*FAM111A*
SELECT	11	rs11214775	0.000796	0.6819	*HTR3B*
SELECT	11	rs11568818	0.01808	0.7962	*MMP7*
PCPT	12	rs10875943	0.02	1.439	*TUBA1C/PRPH*
SELECT	12	rs1270884	0.04893	0.8222	*TBX5*
SELECT	12	rs80130819	0.01114	0.6233	N/A
SELECT	14	rs8014671	0.03711	1.224	N/A
SELECT	17	rs1859962	0.004161	1.328	N/A
SELECT	17	rs138213197	0.005718	NA	N/A
SELECT	17	rs1859962	0.009122	1.3	N/A
SELECT	20	rs6062509	0.001863	0.71	*ZGPAT*
SELECT	22	rs5759167	0.02545	0.8011	N/A
SELECT	22	rs9623117	0.03146	0.7666	N/A
SELECT	23	rs5919432	0.003371	1.662	*AR*
SELECT	23	rs5945619	3.75E-05	1.784	*NUDT11*
SELECT	23	rs2405942	0.01501	0.6641	*SHROOM2*

**Table 6:** Polygenic risk score analyses results. P value corresponding to beta estimate. Weighted polygenic risk score weighted by log-odds ratio

	Beta	*P*
Unweighted polygenic risk score PCPT		
SNP score category 2	0.45	0.001
SNP score category 3	0.67	3E−06
SNP score category 4	1.01	8E−15
Drug	0.09	0.33
Weighted polygenic risk score PCPT		
SNP score category 2	0.4	0.01
SNP score category 3	0.57	0.00002
SNP score category 4	0.79	1E−08
Drug	0.1	0.29
Unweighted polygenic risk score SELECT		
SNP score category 2	0.13	0.6
SNP score category 3	0.08	0.68
SNP score category 4	0.2	0.31
Selenium	0.21	0.28
Vitamin E	0.13	0.5
Selenium & vitamin E	0.08	0.7
Weighted polygenic risk score SELECT		
SNP score category 2	0.23	0.04
SNP score category 3	0.23	0.04
SNP score category 4	0.52	5E−08
Selenium	−0.04	0.66
Vitamin E	0.07	0.48
Selenium & vitamin E	0	0.65

**Fig. 4:**
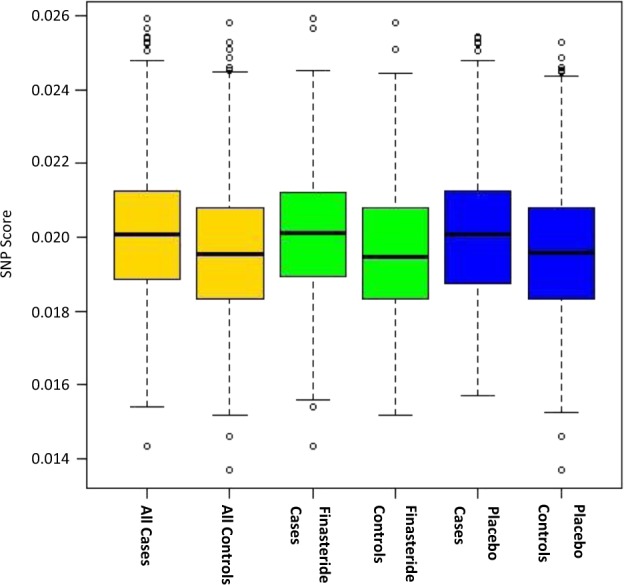
Boxplot showing the weighted polygenic risk score which is higher in cases than controls and is statistically significant (*P* < 0.05) in the PCPT trial

**Fig. 5:**
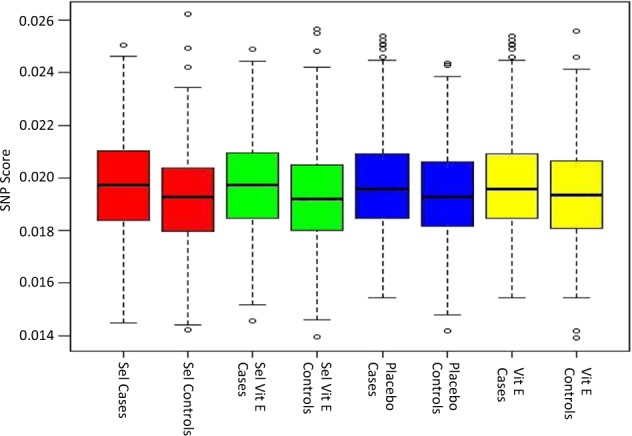
Boxplot showing the weighted polygenic risk score which is higher in cases than controls and is statistically significant (*P* < 0.05) in the SELECT trial

In the PCPT trial, the use of drug intervention did predict the outcome of developing high-grade prostate cancer, but the chemopreventions in the SELECT trial were null (Table 7). The weighted polygenic risk score was consistently higher in cases than controls in all groups and this was also statistically significant (*P* < 0.05) in both PCPT and SELECT trials (Figs 4 and 5). Men in the study who had a first degree relative with prostate cancer had a higher polygenic SNP score in both studies, but this was not statistically significant (*P* > 0.05).

**Table 7:** Showing weighted polygenic risk score, trial drug and family history predicting Gleason score

Weighted SNP score to predict Gleason Cat (HG/LG) using drug and family history	Beta	*P*
PCPT		
SNP score category 2	0.44	0.11
SNP score category 3	0.41	0.1
SNP score category 4	0.14	0.57
Drug	0.69	0.0000007
Family history	−0.02	0.9
SELECT		
SNP score category 2	−0.56	0.003
SNP score category 3	−0.17	0.33
SNP score category 4	−0.28	0.08
Selenium	0	1
Vitamin E	0.03	0.84
Selenium & vitamin E	−0.09	0.6
Family history	−0.15	0.23

## Discussion

This study confirmed that men in both the PCPT and SELECT trials who developed prostate cancer had a higher polygenic risk score than men who did not develop prostate cancer. The study found no evidence that a high polygenic risk score in combination with other risk factors such as FHx could predict if the drug interventions could reduce prostate cancer incidence or development of high grade prostate cancer. Some individual SNPs were detected to predict the likelihood of developing cancer in the individual study arms but these analyses were limited by the power. Rare variants such as those found in sequencing of *BRCA1* and *BRCA2* were not included in this analysis as they require sequencing rather than genotyping in larger cohorts using different statistical analyses. This study was limited to prostate cancer risk associated with common variants of European ancestry and is not useable in other ethnic groups.

The results of the PCPT trial showed a 24.8% reduction in prostate cancer, but the chemoprevention in the SELECT trial dud bit reduce prostate cancer incidence [8, 9]. Prostate cancer SNPs are mostly found in intronic regions of genes, and therefore functionally it is not clear how these SNPs increase prostate cancer risk.

Genetic variations in the pathways in which the chemoprevention agents act may influence the efficacy of the agent. Finasteride acts by inhibiting the enzyme 5α reductase which is mediated by genes of the *SRF5A* family [31]. Polymorphisms in the *SRFA* genes have been reported which affect the efficacy of Finasteride [32, 33]. Genetic variations in the selenoprotein genes impact on plasma selenium levels, and recent evidence suggests that this may be associated with locally advanced or aggressive prostate cancer [14]. Variations in vitamin E levels may also be modified by genetic variations in vitamin E related genes and an association between these and a lower incidence of prostate cancer has been found [12]. If the above mechanisms that affect the outcome of these chemoprevention agents were in common functional pathways with the prostate cancer risk SNPs, men in the highest polygenic risk score would be more likely to see some of the above benefits from chemoprevention. Many of the pathways in which these genetic variants affect drug efficacy remain unknown, which therefore could be accounting for the null results of this study.

In the single SNP association for the development of prostate cancer a number of SNPs pass the significance level. Functionally it is not clear how these associations are linked but there are potentially some interesting regions. One interesting SNP which was statistically significant in the Finasteride arm of the PCPT trial was rs4430796 which resides near the gene *HNF1B* [34]. This SNP is in strong linkage disequilibrium with SNP rs757210. It has been reported that inheriting the risk allele for one of these SNPs increases the likelihood of developing prostate cancer (OR = 1.22, 95% CI 1.15–1.30; *P* = 1.4 × 10^–11^), but reduces the risk of developing type 2 diabetes (OR = 0.91, 95% CI 0.88–0.93; *P* = 8 × 10^–10^) [35]. The association between the phenotypes of type 2 diabetes and prostate cancer was further investigated in the PCPT trial. This showed that type 2 diabetes was associated with a 47% reduction of low grade prostate cancer and 28% reduction of high grade prostate cancer [36]. When the authors looked at the association of obesity and prostate cancer they found that increased obesity reduced low grade prostate cancer but increased high grade prostate cancer [36].The authors also showed that there was no correlation between obesity and type 2 diabetes, suggesting an independent pathway in which diabetes protects against prostate cancer. Our analysis supports these data which shows that diabetes incidence is lower in men who develop prostate cancer in the placebo arm and inherit one of the SNPs near *HNF1B*. However this association is not seen in men who have Finasteride who have higher rates of type 2 diabetes and could possibly suggest a metabolic interaction between the drug and the SNP.

On analyses of the SNP association with high grade and low grade Gleason score a number of SNPs show an association with Gleason grade in the individual trial arms. An example in the Finasteride arm of the PCPT, SNP rs7127900 showed a statistically significant reduction in the odds ratio of developing high grade prostate cancer (*P* = 0.04, OR = 0.67). Interestingly SNP rs7127900 has been shown to have a biological interaction with insulin-like-growth-factor-2 [37]. Analyses from the PCPT trial have shown that serum levels of IGF were not correlated with prostate cancer development; however, men who have high levels of IGF are more likely to be on anti-diabetic drugs such as metformin which have shown to have anti-cancer properties [38]. In the SELECT trial SNP rs12621278 in the vitamin E arm was also associated with a reduced odds ratio of developing high grade prostate cancer. The SNP rs12621278 resides near the integrin gene *ITGA6* which has been shown to be associated with prostate cancer progression and development [39]. Vitamin E could possibly be interacting with this pathway [40].

So far 33% of common genetic variants that predict the familial risk of prostate cancer have been discovered [16]. Men in the top 1% risk distribution have a 5.7-fold relative risk of developing prostate cancer compared with the average population being profiled [24]. The National Institutes of Health GAME-ON initiative has discovered a further 63 new prostate cancer susceptibility SNPs. Further work needs to be performed to understand if these new SNPs will help understand the biological rationale for chemoprevention [28].

In summary this work has shown that a high polygenic risk score can predict the development of prostate cancer but there is no interaction with chemoprevention agents such as finasteride and selenium/vitamin E. This is an important null finding as population risk stratification will be undertaken in coming years for disease detection and prevention strategies. There is therefore no evidence from these results that certain risk groups are individually more likely to benefit from these two types of chemoprevention and other types of agent will need to be tested to try to reduce risk of high grade cancers in men with higher polygenic risk scores.

## Supplementary information

Supplemental Table 2

Supplementary Table 1
