# Supplementary material for: Germline genetic variation in prostate susceptibility does not predict outcomes in the chemoprevention trials PCPT and SELECT
Source: Prostate Cancer Prostatic Dis. 2019 Nov 27;23(2):333–42. doi: 10.1038/s41391-019-0181-y (PMC7237354; doi:10.1038/s41391-019-0181-y)
Supplement: Supplementary file 1 — Supplemental Table 2 [file 41391_2019_181_MOESM1_ESM.docx]

Supplementary Table 2- showing the 100 SNP susceptibility loci. rs7153648 and rs1775148 did not design and therefore are not included in analysis. Nearby genes relates to genes +/- 500kb from the SNP.

| Locus | SNP | Effect allele | Reference allele | Per allele OR* | Nearby genes | Reference | | |
| --- | --- | --- | --- | --- | --- | --- | --- | --- |
| 1q21 | rs1218582 | A | G | 1.06 (1.03-1.09) |  | | (24) |  |
| 1q32 | rs4245739 | A | A | 1.10 (1.05-1.14) |  | | (24) |  |
| 1q21 | rs17599629 | A | G | 1.10 (1.07-1.13) | *GOLPH3L* | | (40) |  |
| 1q32 | rs1775148 | T | C | 1.06 (1.03-1.08) | *SLC41A1* | | (40) |  |
| 1p35 | rs636291 | G | A | 1.18(1.12-1.24) | *PEX14* | | (40) |  |
| 2p21 | rs1465618 | C | T | 1.07 (1.04-1.11) | *THADA* | | (41) |  |
| 2q31 | rs12621278 | A | A | 1.33 (1.25-1.43) | *ITGA6* | | (41) |  |
| 2p11 | rs10187424 | T | T | 1.09 (1.06-1.12) |  | | (42) |  |
| 2q37 | rs7584330 | A | G | 1.06 (1.02-1.09) | *MLPH* | | (42, 43) |  |
| 2p25 | rs11902236 | C | T | 1.07 (1.03-1.10) |  | | (24) |  |
| 2q37 | rs3771570 | C | T | 1.12 (1.08-1.17) | *FARP2* | | (24) |  |
| 2p25 | rs9287719 | T | C | 1.07 (1.04-1.09) | *NOL10* | | (40) |  |
| 2p15 | rs721048 | G | A | 1.12 (1.07-1.16) |  | | (44) |  |
| 2p24 | rs13385191 | G | G | 1.15 (1.10-1.21) | *C2orf43* | | (45) |  |
| 3p12 | rs2660753 | C | T | 1.13 (1.08-1.19) |  | | (41) |  |
| 3q23 | rs6763931 | G | A | 1.04 (1.01-1.07) | *ZBTB38* | | (42) |  |
| 3q26 | rs10936632 | A | A | 1.11 (1.08-1.14) |  | | (42) |  |
| 3q13 | rs7611694 | A | A | 1.10 (1.08-1.14) |  | | (24) |  |
| 3q21 | rs10934853 | C | A | 1.12 (1.08-1.16) | *EEFSEC* | | (46) |  |
| 4q22 | rs17021918 | C | C | 1.14 (1.10-1.18) | *PDLIM5* | | (41) |  |
| 4q22 | rs12500426 | C | A | 1.10 (1.06-1.13) | *PDLIM5* | | (41) |  |
| 4q24 | rs7679673 | C | C | 1.15 (1.11-1.18) | *TET2* | | (41) |  |
| 4q13 | rs1894292 | G | G | 1.10 (1.06-1.12) |  | | (24) |  |
| 4q13 | rs10009409 | C | T | 1.09 (1.06-1.12) |  | | (40) |  |
| 5p12 | rs2121875 | A | C | 1.05 (1.02-1.08) | *FGF10* | | (42) |  |
| 5p15 | rs2242652 | C | C | 1.15 (1.11-1.19) | *TERT* | | (42, 47) |  |
| 5p15 | rs2853676 | C | T | 1.09 (1.05-1.12) | *TERT* | | (47) |  |
| 5p15 | rs2736107 | C | T | 1.12 (1.08-1.15) | *TERT* | | (47) |  |
| 5p15 | rs13190087 | A | C | 1.20 (1.12-1.29) | *TERT* | | (47) |  |
| 5q35 | rs6869841 | C | T | 1.07 (1.04-1.11) |  | | (24) |  |
| 6q25 | rs9364554 | C | T | 1.10 (1.06-1.14) | *SLC22A3* | | (41) |  |
| 6p21 | rs130067 | T | G | 1.05 (1.02-1.09) | *CCHCR1* | | (42) |  |
| 6p21 | rs3096702 | G | A | 1.07 (1.04-1.10) | *NOTCH4* | | (24) |  |
| 6q21 | rs2273669 | A | G | 1.07 (1.03-1.11) |  | | (24) |  |
| 6q25 | rs1933488 | A | A | 1.12 (1.09-1.15) |  | | (24) |  |
| 6p21 | rs3129859 | G | G | 1.08 (1.06-1.11) | *HLA-DRA* | | (40) |  |
| 6p22 | rs7767188 | G | A | 1.08 (1.06-1.11) | *TRIM31* | | (40) |  |
| 6p24 | rs4713266 | C | C | 1.08 (1.04-1.09) | *NEDD9* | | (40) |  |
| 6q14 | rs9443189 | A | G | 1.07 (1.04-1.11) | *MYO6* | | (40) |  |
| 7q21 | rs6465657 | T | C | 1.10 (1.07-1.13) | *LMTK2* | | (41) |  |
| 7p15 | rs12155172 | G | A | 1.11 (1.07-1.15) | *SP8* | | (24) |  |
| 7p12 | rs56232506 | G | A | 1.07 (1.05-1.09) | *TNS3* | | (40) |  |
| 7p15 | rs10486567 | G | G | 1.18 (1.12-1.22) | *JAZF1* | | (48) |  |
| 8p21 | rs2928679 | G | A | 1.04 (1.01-1.07) | *SLC25A37* | | (41) |  |
| 8p21 | rs1512268 | C | T | 1.13 (1.10-1.17) | *NKX3.1* | | (41) |  |
| 8q24 |  rs10086908 | T | T | 1.15 (1.06-1.23) |  | | (49) |  |
| 8q24 | rs12543663 | A | C | 1.08 (1.00-1.16) |  | | (49) |  |
| 8q24 |  rs620861 | C | C | 1.11 (1.04-1.19) |  | | (49) |  |
| 8p21 | rs11135910 | C | T | 1.11 (1.07-1.16) | *EBF2* | | (24) |  |
| 8q24 | rs1447295 | C | A | 1.42 (1.35-1.49) |  | | (50) |  |
| 8q24 | rs6983267 | G | G | 1.22 (1.19-1.27) |  | | (51) |  |
| 8q24 | rs16901979 | C | A | 1.55 (1.43-1.68) |  | | (52) |  |
| 9p21 | rs17694493 | C | G | 1.10 (1.06-1.13) | *CDKN2B-AS1* | | (40) |  |
| 9q33 | rs1571801 | G | T | 1.27 (1.10-1.48) | *DAB2IP* | | (53) |  |
| 10q11 | rs10993994 | C | T | 1.24 (1.20-1.28) | *MSMB* | | (41) |  |
| 10q24 | rs3850699 | A | A | 1.10 (1.06-1.12) | *TRIM8* | | (24) |  |
| 10q11 | rs76934034 | T | T | 1.14 (1.10-1.18) | *Mar-08* | | (40) |  |
| 10q26 | rs4962416 | T | C | 1.04 (1.00-1.09) | *CTBP2* | | (48) |  |
| 11q13 | rs7931342 | G | G | 1.20 (1.16-1.23) |  | | (41) |  |
| 11p15 | rs7127900 | G | A | 1.23 (1.18-1.28) |  | | (41) |  |
| 11q22 | rs11568818 | T | T | 1.10 (1.06-1.14) | *MMP7* | | (24) |  |
| 11q23 | rs11214775 | G | G | 1.08 (1.05-1.11) | *HTR3B* | | (40) |  |
| 12q13 | rs10875943 | T | C | 1.07 (1.04-1.10) |  | | (42) |  |
| 12q24 | rs1270884 | G | A | 1.07 (1.04-1.10) | *TBX5* | | (24) |  |
| 12q13 | rs80130819 | A | A | 1.12 (1.08-1.18) |  | | (40) |  |
| 12q13 | rs902774 | G | A | 1.17 (1.11-1.24) | *KRT8* | | (43) |  |
| 14q22 | rs8008270 | C | C | 1.12 (1.08-1.16) | *FERMT2* | | (24) |  |
| 14q24 | rs7141529 | T | C | 1.09 (1.06-1.12) | *RAD51B* | | (24) |  |
| 14q24 | rs8014671 | G | G | 1.08 (1.05-1.10) |  | | (40) |  |
| 14q23 | rs7153648 | G | C | 1.09 (1.04-1.13) | *SIX1* | | (40) |  |
| 16q22 | rs12051443 | G | A | 1.06 (1.03-1.08) | *PHLPP2* | | (40) |  |
| 17p13 | rs684232 | T | C | 1.10 (1.07-1.14) |  | | (24) |  |
| 17q21 | rs11650494 | G | A | 1.15 (1.09-1.22) |  | | (24) |  |
| 17q12 | rs4430796 | A | A | 1.22 (1.19-1.27) | *HNF1B* | | (54) |  |
| 17q12 | rs11649743 | G | G | 1.14 (1.10-1.19) | *HNF1B* | | (55) |  |
| 17q24 | rs1859962 | T | G | 1.19 (1.14-1.23) |  | | (54, 56) |  |
| 18q23 | rs7241993 | C | C | 1.09 (1.05-1.12) |  | | (24) |  |
| 19q13 | rs2735839 | G | G | 1.23 (1.18-1.30) | *KLK2/3* | | (41) |  |
| 19q13 | rs11672691 | G | A | 1.08 (1.05-1.12) | *PCAT19* | | (57, 58) |  |
| 19q13 | rs8102476 | C | C | 1.12 (1.08-1.15) |  | | (46) |  |
| 20q13 | rs2427345 | C | C | 1.06 (1.03-1.10) |  | | (24) |  |
| 20q13 | rs6062509 | T | T | 1.12 (1.09-1.16) | *ZGPAT* | | (24) |  |
| 20q13 | rs12480328 | T | T | 1.14 (1.08-1.18) | *ADNP* | | (40) |  |
| 21q22 | rs1041449 | A | G | 1.06 (1.04-1.09) | *TMPRSS2* | | (40) |  |
| 22q13 | rs5759167 | G | G | 1.19 (1.15-1.22) |  | | (41) |  |
| 22q11 | rs2238776 | G | G | 1.09 (1.06-1.12) | *TBX1* | | (40) |  |
| 22q13 | rs9623117 | T | C | 1.11 (1.04-1.19) | *TNRC6B* | | (59) |  |
| Xp11 | rs5945619 | T | C | 1.28 (1.21-1.35) | *NUDT11* | | (41) |  |
| Xq12 | rs5919432 | T | C | 1.06 (1.02-1.12) | *AR* | | (42) |  |
| Xp22 | rs2405942 | A | A | 1.14 (1.09-1.20) |  | | (24) |  |
| Xp11 | rs2807031 | T | C | 1.07 (1.04-1.09) | *XAGE3* | | (40) |  |
