# Supplementary material for: Germline genetic variation in prostate susceptibility does not predict outcomes in the chemoprevention trials PCPT and SELECT
Source: Prostate Cancer Prostatic Dis. 2019 Nov 27;23(2):333–42. doi: 10.1038/s41391-019-0181-y (PMC7237354; doi:10.1038/s41391-019-0181-y)
Supplement: Supplementary file 2 — Supplementary Table 1 [file 41391_2019_181_MOESM2_ESM.docx]

| **Locus** | **SNP** | **Effect allele** | **Reference allele** | **Per allele OR*** | **OR** | **EAF** | **PCPT power** | **SELECT power** |
| --- | --- | --- | --- | --- | --- | --- | --- | --- |
| **1q21** | rs1218582 | A | G | 1.06 (1.03-1.09) | 1.04 | 0.45 | 0.10 | 0.13 |
| **1q32** | rs4245739 | A | A | 1.10 (1.05-1.14) | 1.10 | 0.74 | 0.27 | 0.39 |
| **1q21** | rs17599629 | A | G | 1.10 (1.07-1.13) | 1.05 | 0.22 | 0.08 | 0.13 |
| **1q32** | rs1775148 | T | C | 1.06 (1.03-1.08) | 1.03 | 0.36 | 0.07 | 0.09 |
| **1p35** | rs636291 | G | A | 1.18(1.12-1.24) | 1.03 | 0.68 | 0.07 | 0.09 |
| **2p21** | rs1465618 | C | T | 1.07 (1.04-1.11) | 1.09 | 0.21 | 0.21 | 0.30 |
| **2q31** | rs12621278 | A | A | 1.33 (1.25-1.43) | 1.26 | 0.94 | 0.39 | 0.56 |
| **2p11** | rs10187424 | T | T | 1.09 (1.06-1.12) | 1.07 | 0.57 | 0.19 | 0.27 |
| **2q37** | rs7584330 | A | G | 1.06 (1.02-1.09) | 1.08 | 0.69 | 0.21 | 0.30 |
| **2p25** | rs11902236 | C | T | 1.07 (1.03-1.10) | 1.03 | 0.27 | 0.07 | 0.08 |
| **2q37** | rs3771570 | C | T | 1.12 (1.08-1.17) | 1.09 | 0.15 | 0.17 | 0.25 |
| **2p25** | rs9287719 | T | C | 1.07 (1.04-1.09) | 1.08 | 0.47 | 0.24 | 0.35 |
| **2p15** | rs721048 | G | A | 1.12 (1.07-1.16) | 1.10 | 0.18 | 0.22 | 0.33 |
| **2p24** | rs13385191 | G | G | 1.15 (1.10-1.21) | 1.04 | 0.20 | 0.08 | 0.10 |
| **3p12** | rs2660753 | C | T | 1.13 (1.08-1.19) | 1.12 | 0.11 | 0.20 | 0.32 |
| **3q23** | rs6763931 | G | A | 1.04 (1.01-1.07) | 1.04 | 0.44 | 0.10 | 0.12 |
| **3q26** | rs10936632 | A | A | 1.11 (1.08-1.14) | 1.10 | 0.50 | 0.34 | 0.49 |
| **3q13** | rs7611694 | A | A | 1.10 (1.08-1.14) | 1.08 | 0.58 | 0.23 | 0.34 |
| **3q21** | rs10934853 | C | A | 1.12 (1.08-1.16) | 1.10 | 0.28 | 0.29 | 0.42 |
| **4q22** | rs17021918 | C | C | 1.14 (1.10-1.18) | 1.07 | 0.65 | 0.18 | 0.26 |
| **4q22** | rs12500426 | C | A | 1.10 (1.06-1.13) | 1.05 | 0.47 | 0.12 | 0.17 |
| **4q24** | rs7679673 | C | C | 1.15 (1.11-1.18) | 1.11 | 0.59 | 0.38 | 0.55 |
| **4q13** | rs1894292 | G | G | 1.10 (1.06-1.12) | 1.05 | 0.52 | 0.10 | 0.13 |
| **4q13** | rs10009409 | C | T | 1.09 (1.06-1.12) | 1.03 | 0.31 | 0.06 | 0.09 |
| **5p12** | rs2121875 | A | C | 1.05 (1.02-1.08) | 1.02 | 0.33 | 0.06 | 0.07 |
| **5p15** | rs2242652 | C | C | 1.15 (1.11-1.19) | 1.19 | 0.79 | 0.61 | 0.80 |
| **5p15** | rs2853676 | C | T | 1.09 (1.05-1.12) | 1.06 | 0.18 | 0.09 | 0.15 |
| **5p15** | rs2736107 | C | T | 1.12 (1.08-1.15) | 1.18 | 0.85 | 0.46 | 0.65 |
| **5p15** | rs13190087 | A | C | 1.20 (1.12-1.29) | 1.04 | 0.20 | 0.08 | 0.10 |
| **5q35** | rs6869841 | C | T | 1.07 (1.04-1.11) | 1.02 | 0.21 | 0.06 | 0.06 |
| **6q25** | rs9364554 | C | T | 1.10 (1.06-1.14) | 1.10 | 0.28 | 0.29 | 0.42 |
| **6p21** | rs130067 | T | G | 1.05 (1.02-1.09) | 1.04 | 0.20 | 0.08 | 0.10 |
| **6p21** | rs3096702 | G | A | 1.07 (1.04-1.10) | 1.05 | 0.37 | 0.12 | 0.16 |
| **6q21** | rs2273669 | A | G | 1.07 (1.03-1.11) | 1.06 | 0.15 | 0.10 | 0.13 |
| **6q25** | rs1933488 | A | A | 1.12 (1.09-1.15) | 1.07 | 0.58 | 0.19 | 0.22 |
| **6p21** | rs3129859 | G | G | 1.08 (1.06-1.11) | 1.05 | 0.37 | 0.12 | 0.16 |
| **6p22** | rs7767188 | G | A | 1.08 (1.06-1.11) | 1.11 | 0.92 | 0.15 | 0.20 |
| **6p24** | rs4713266 | C | C | 1.08 (1.04-1.09) | 1.04 | 0.52 | 0.10 | 0.13 |
| **6q14** | rs9443189 | A | G | 1.07 (1.04-1.11) | 1.07 | 0.86 | 0.12 | 0.16 |
| **7q21** | rs6465657 | T | C | 1.10 (1.07-1.13) | 1.10 | 0.46 | 0.34 | 0.49 |
| **7p15** | rs12155172 | G | A | 1.11 (1.07-1.15) | 1.08 | 0.22 | 0.18 | 0.26 |
| **7p12** | rs56232506 | G | A | 1.07 (1.05-1.09) | 1.04 | 0.45 | 0.10 | 0.13 |
| **7p15** | rs10486567 | G | G | 1.18 (1.12-1.22) | 1.15 | 0.76 | 0.47 | 0.66 |
| **8p21** | rs2928679 | G | A | 1.04 (1.01-1.07) | 1.05 | 0.44 | 0.12 | 0.17 |
| **8p21** | rs1512268 | C | T | 1.13 (1.10-1.17) | 1.14 | 0.43 | 0.56 | 0.76 |
| **8q24** | rs10086908 | T | T | 1.15 (1.06-1.23) | 1.14 | 0.43 | 0.56 | 0.76 |
| **8q24** | rs12543663 | A | C | 1.08 (1.00-1.16) | 1.11 | 0.29 | 0.34 | 0.50 |
| **8q24** | rs620861 | C | C | 1.11 (1.04-1.19) | 1.11 | 0.29 | 0.34 | 0.50 |
| **8p21** | rs11135910 | C | T | 1.11 (1.07-1.16) | 1.06 | 0.15 | 0.10 | 0.14 |
| **8q24** | rs1447295 | C | A | 1.42 (1.35-1.49) | 1.43 | 0.10 | 0.96 | 1.00 |
| **8q24** | rs6983267 | G | G | 1.22 (1.19-1.27) | 1.21 | 0.51 | 0.87 | 0.97 |
| **8q24** | rs16901979 | C | A | 1.55 (1.43-1.68) | 1.51 | 0.03 | 0.71 | 0.89 |
| **9p21** | rs17694493 | C | G | 1.10 (1.06-1.13) | 1.05 | 0.14 | 0.08 | 0.11 |
| **9q33** | rs1571801 | G | T | 1.27 (1.10-1.48) | 1.01 | 0.27 | 0.05 | 0.05 |
| **10q11** | rs10993994 | C | T | 1.24 (1.20-1.28) | 1.24 | 0.38 | 0.91 | 0.99 |
| **10q24** | rs3850699 | A | A | 1.10 (1.06-1.12) | 1.06 | 0.70 | 0.14 | 0.19 |
| **10q11** | rs76934034 | T | T | 1.14 (1.10-1.18) | 1.11 | 0.92 | 0.15 | 0.20 |
| **10q26** | rs4962416 | T | C | 1.04 (1.00-1.09) | 1.06 | 0.27 | 0.13 | 0.19 |
| **11q13** | rs7931342 | G | G | 1.20 (1.16-1.23) | 1.16 | 0.51 | 0.67 | 0.85 |
| **11p15** | rs7127900 | G | A | 1.23 (1.18-1.28) | 1.18 | 0.20 | 0.59 | 0.79 |
| **11q22** | rs11568818 | T | T | 1.10 (1.06-1.14) | 1.08 | 0.55 | 0.23 | 0.34 |
| **11q23** | rs11214775 | G | G | 1.08 (1.05-1.11) | 1.08 | 0.70 | 0.20 | 0.24 |
| **12q13** | rs10875943 | T | C | 1.07 (1.04-1.10) | 1.06 | 0.29 | 0.14 | 0.19 |
| **12q24** | rs1270884 | G | A | 1.07 (1.04-1.10) | 1.07 | 0.48 | 0.19 | 0.28 |
| **12q13** | rs80130819 | A | A | 1.12 (1.08-1.18) | 1.10 | 0.91 | 0.14 | 0.19 |
| **12q13** | rs902774 | G | A | 1.17 (1.11-1.24) | 1.13 | 0.15 | 0.30 | 0.44 |
| **14q22** | rs8008270 | C | C | 1.12 (1.08-1.16) | 1.07 | 0.81 | 0.14 | 0.19 |
| **14q24** | rs7141529 | T | C | 1.09 (1.06-1.12) | 1.04 | 0.50 | 0.10 | 0.13 |
| **14q24** | rs8014671 | G | G | 1.08 (1.05-1.10) | 1.03 | 0.58 | 0.08 | 0.09 |
| **14q23** | rs7153648 | G | C | 1.09 (1.04-1.13) | 1.02 | 0.08 | 0.05 | 0.06 |
| **16q22** | rs12051443 | G | A | 1.06 (1.03-1.08) | 1.02 | 0.34 | 0.06 | 0.07 |
| **17p13** | rs684232 | T | C | 1.10 (1.07-1.14) | 1.09 | 0.35 | 0.27 | 0.39 |
| **17q21** | rs11650494 | G | A | 1.15 (1.09-1.22) | 1.09 | 0.08 | 0.12 | 0.16 |
| **17q12** | rs4430796 | A | A | 1.22 (1.19-1.27) | 1.22 | 0.52 | 0.89 | 0.98 |
| **17q12** | rs11649743 | G | G | 1.14 (1.10-1.19) | 1.13 | 0.81 | 0.33 | 0.48 |
| **17q24** | rs1859962 | T | G | 1.19 (1.14-1.23) | 1.17 | 0.48 | 0.67 | 0.89 |
| **18q23** | rs7241993 | C | C | 1.09 (1.05-1.12) | 1.08 | 0.69 | 0.21 | 0.30 |
| **19q13** | rs2735839 | G | G | 1.23 (1.18-1.30) | 1.18 | 0.85 | 0.46 | 0.65 |
| **19q13** | rs11672691 | G | A | 1.08 (1.05-1.12) | 1.09 | 0.74 | 0.27 | 0.39 |
| **19q13** | rs8102476 | C | C | 1.12 (1.08-1.15) | 1.11 | 0.54 | 0.39 | 0.56 |
| **20q13** | rs2427345 | C | C | 1.06 (1.03-1.10) | 1.03 | 0.62 | 0.07 | 0.09 |
| **20q13** | rs6062509 | T | T | 1.12 (1.09-1.16) | 1.06 | 0.70 | 0.14 | 0.19 |
| **20q13** | rs12480328 | T | T | 1.14 (1.08-1.18) | 1.11 | 0.93 | 0.13 | 0.19 |
| **21q22** | rs1041449 | A | G | 1.06 (1.04-1.09) | 1.04 | 0.43 | 0.10 | 0.12 |
| **22q13** | rs5759167 | G | G | 1.19 (1.15-1.22) | 1.17 | 0.50 | 0.72 | 0.89 |
| **22q11** | rs2238776 | G | G | 1.09 (1.06-1.12) | 1.04 | 0.80 | 0.08 | 0.10 |
| **22q13** | rs9623117 | T | C | 1.11 (1.04-1.19) | 1.02 | 0.44 | 0.06 | 0.07 |
| **Xp11** | rs5945619 | T | C | 1.28 (1.21-1.35) | 1.11 | 0.36 | 0.37 | 0.54 |
| **Xq12** | rs5919432 | T | C | 1.06 (1.02-1.12) | 1.04 | 0.80 | 0.08 | 0.10 |
| **Xp22** | rs2405942 | A | A | 1.14 (1.09-1.20) | 1.04 | 0.78 | 0.08 | 0.08 |
| **Xp11** | rs2807031 | T | C | 1.07 (1.04-1.09) | 1.06 | 0.18 | 0.09 | 0.15 |

Supplementary Table 1: Power Calculation for SNPs used OR = Odds Ratio, EF = Effect allele – obtained from OncoArray main paper in prostate cancer. Significance α = 0.05
